# Muscles and Central Neural Networks Involved in Breathing: State of the Art

**DOI:** 10.7759/cureus.80599

**Published:** 2025-03-15

**Authors:** Bruno Bordoni, Allan R Escher

**Affiliations:** 1 Physical Medicine and Rehabilitation, Foundation Don Carlo Gnocchi, Milan, ITA; 2 Oncologic Sciences, University of South Florida Morsani College of Medicine, Tampa, USA; 3 Anesthesiology/Pain Medicine, H. Lee Moffitt Cancer Center and Research Institute, Tampa, USA

**Keywords:** breathing, chf, copd, diaphragm, osas, physiotherapy, respiration

## Abstract

Breathing is a systemic act, which involves not only the lungs, but the entire body system. To have a comprehensive clinical picture, it is necessary to have all the patient's data; from this assumption, we can affirm that it is necessary to know all the muscles involved in breathing to understand how to obtain a comprehensive approach for the care and treatment of the patient to improve respiratory capacity. The text reviews the efferent connections of the respiratory centers and cites all the muscles that are involved in the mechanism of breathing and that are controlled and managed by the respiratory centers, starting from the muscular description of the cranial area, the bucco-cervical area, the cervicothoracic area, and the thoracic area. Knowing the function of the respiratory accessory muscles allows us to obtain, in some clinical cases, valuable data that can prove predictive of the diagnostic path of the pathology.

This is the first article in the literature, to the authors' knowledge, that attempts to list and include in a single text all the muscles directly or indirectly involved in breathing. The goal of this narrative review article is to remind clinicians and researchers involved in the study of different muscular respiratory responses that we need to analyze and work all the skeletal musculature involved in breathing to better understand what happens in the pathological or physiological phases during breathing. This step will allow us to better individualize the therapeutic and training approach for healthy subjects.

## Introduction and background

Breathing is a systemic process that involves not only the lungs but the entire body [1]. In a healthy adult at rest, the respiratory rate is 12-20 breaths per minute on average; in a child the rate can vary between 26 breaths per minute (from the first two years of life), up to 44 at birth [2]. The inhalation/exhalation mode affects the biochemical aspect and metabolic responses, interacting with a variety of receptors (chemoreceptors from the carotid body, proprioceptors from the rib cage), passing from the air in the lungs to cellular phosphorylation. Metabolic reactions occur in the mitochondria, the main by-product of which is carbon dioxide, which will be directed into the venous blood, up to the lungs. Carbon dioxide arrives and diffuses through the alveoli, to be dispersed in the exhaled air [2]. The biochemical balance of breathing (respiratory quotient, or respiratory ratio) is a physiological relationship between the volume of oxygen consumed and the volume of carbon dioxide produced. The respiratory quotient is a dimensionless number to correctly frame the type of basal metabolism of a person; to do this, a spirometer is used, which is placed on the tissue or mouth [3]. The values considered normal are 200 mL/minute of carbon dioxide and 250 mL/minute of oxygen consumed (200/250 = 0.8). It is calculated for specific substrates, such as organic acids, sugars, and protein structures; in particular, carbohydrates are consumed by aerobic respiration, with an equal ratio of CO2 release and O2 utilization. The respiratory quotient for lipids, proteins, and anaerobes will be 0.7, 0.8, and zero, respectively. The result of a used mixture of different substrates, will give a collective value of 0.8 [3].

An alteration in the respiratory rate implies the presence of a pathology. An increase in the blood concentration of hydrogen ions (acidosis or acidemia), with a decrease in pH, indicates a systemic anaerobic metabolic environment, where the patient increases the respiratory rate; in a very alkaline environment (pH over 7.45), the respiratory rate decreases [2]. In the presence of chronic pathologies, such as obesity, obstructive sleep apnea syndrome (OSAS), chronic heart failure (CHF), chronic obstructive pulmonary disease (COPD), as well as the presence of chronic altered emotions or psychic states (depression, anxiety, stress), the respiratory rate changes in a non-physiological way [4-8]. Patients who show chronicity related to pain and functional limitation of the body structure, such as chronic nonspecific low back pain (CNSLBP), chronic neck pain, chronic painful temporomandibular disorder, chronic ankle instability and chronic pelvic pain, present a contractile alteration of the diaphragm muscle [9-13].

The presence of pancreatic ductal adenocarcinoma (PDAC) generates an inflammatory environment that alters the phenotype, morphology, and function of the diaphragm muscle and respiratory accessory districts; this latter alteration leads to physiopathological changes in systemic metabolism, causing tumor cachexia [14].

Patients with pulmonary hypertension undergo adaptations of the pulmonary vascular system and respiratory muscles, and limbs; symptoms of exercise intolerance are linked to pathological alterations in the vascularization of the respiratory muscles. In fact, during exercise, blood flow to the (already weak) respiratory muscles increases, reducing blood volume to the skeletal muscles of the limbs and trunk [15].

An alteration of the respiratory musculature negatively affects systemic health parameters, such as cardiac and pulmonary function, muscular function, intestinal function, cognition, pain tolerance, and other bodily functions [1,16-19].

When the clinician assesses the need to send the patient to a rehabilitation setting to improve respiratory function, for various reasons that negatively impact the function of the respiratory system, a respiratory physiotherapy process is set up. Access to a rehabilitation regimen is not exclusive to patients with COPD, but patients with different clinical histories can access it, such as oncology patients with chronic pain and respiratory functional depression [20-24].

Literature mainly directs research/treatment toward improving lung function through specific instruments that provide resistance during inhalation and exhalation, for training the diaphragmatic muscle. What is missing in all studies, to the best of the authors' knowledge, is to take into consideration in the rehabilitation process (treatment and/or evaluation) all the muscles that influence the respiratory act, which are managed by the respiratory centers. These are the muscles that create the pressures necessary for the functioning of the lungs, and not vice versa. This narrative review cites and describes the functions of all the muscles involved in breathing, since, probably, by evaluating and treating all the muscles of the respiratory system, better results could be obtained in the rehabilitation process. This reflection will need to be explored with further research, which is currently absent.

This narrative review is divided into sections, the first of which highlights the most significant neural connections for the management of breathing; the following paragraphs list the accessory muscles of the different body areas: skull area, bucco-cervical area, cervico-thoracic area, thoracic area, and pelvic area. This is a subdivision into body areas for ease of writing the text, since, in the reality of the living being, all the main and accessory respiratory muscles are managed and controlled by the same neural respiratory centers in a perfect continuum.

## Review

Central respiratory pattern generator and other neural areas

All respiratory muscles innervated by cranial and spinal nerves are governed by the central respiratory pattern generator (CRPG) [25-28]. The control of the complex mechanisms of breathing is always subconscious, since even if a person decides to manage the respiratory rhythm or the amount of air inhaled or exhaled, he or she will never know the exact number of motor units involved, as well as the correct amount of Hertz needed for each individual muscle fiber, or it will not be possible to voluntarily know the exact number of neurons involved in the excitation or inhibition of certain spinal areas or of the central nervous system [29].

CRPG controls respiratory rhythms and the neural network connected to it; its main nuclei are found at the level of the pons and the medulla oblongata, while the direct and indirect neural network for respiratory control is found in different brain and spinal areas. The neural complex fundamental for the initiation of inspiration is the pre-Bötzinger complex (preBötzC), located in the medullary area. The neurons of the latter are interconnected with the neurons responsible for expiration, with a number of neurons of approximately 3000 on each side; it contains interneurons sensitive to the presence/absence of somatostatin (neuropeptide), which can send excitatory or inhibitory signals [30]. The activation of excitatory and inhibitory interneurons and the variable amount of somatostatin creates a rhythm, fundamental for respiration. Furthermore, the proper neurons of the preBötzC area can send excitatory signals to the phrenic respiratory motor nuclei and, at the same time, send inhibitory signals to activate the cardiac parasympathetic neuron system and excitatory signals to manage the sympathetic vasomotor neurons; in this way, breathing is connected to and influences cardiovascular rhythms [31].

preBötzC is found in the area or column of the ventral respiratory group (VRG), within which we can find in the central area the nucleus ambiguus and the peri-ambiguous area (with inspiratory functions), Bötzinger complex (BötzC) with expiratory functions; the rostral respiratory group (rVRG) containing the retrotrapezoid nucleus (for prolonged inspiration and forced expiration) and the parafacial respiratory group (for inspiration), with functions of modulating the respiratory frequency; the caudal VRG (cVRG) where the retro-ambiguous nucleus resides (expiratory functions); finally, we find the dorsal respiratory group (DRG) where the nucleus of the solitary tract (NTS) resides, which is fundamental for controlling the quality and quantity of breathing, managing the afferents from different viscera; the Kölliker-Fuse and parabrachial nuclei are subareas of VRG that regulate the shutdown of the inspiratory and expiratory phases [28,32,33].

CRPG is essential for breathing, but also for controlling multiple related functions such as speaking, singing, coughing, sneezing, swallowing, as well as other functions described in detail in previous works [1,6,19,27].

The post-inspiratory complex (PiCo) (ventromedial medulla) is activated to manage the post-inspiratory action; it is mainly constituted by excitatory cholinergic neurons. PiCo interacts with some CRPG areas, such as preBötzC and retrotrapezoid nucleus [34].

Locus coeruleus (brainstem) contains noradrenergic neurons with direct neural connections with preBötzC. When there is a change in carbon dioxide (hypoxia or hypercapnia), locus coeruleus intervenes to modulate the amount of inspiration and expiration [35]. In addition, it sends several efferents, including the phrenic nuclei, Kölliker-Fuse and parabrachial nuclei, and NTS [29].

Raphe nuclei caudal (brainstem) is rich in serotoninergic neurons; in the presence of severe hypoxia it is activated to stimulate or inhibit parabrachial nuclei, retrotrapezoid nucleus, locus coeruleus. The behavior of this structure will depend on the circumstances that led to its activation [29,36].

The lateral hypothalamus is a source of orexin, a neuropeptide able to stimulate the phrenic nerve (and the hypoglossal nerve), and to influence the behavior of other respiratory areas, such as preBötzC, Kölliker-Fuse nuclei, raphe caudal nuclei, retrotrapezoid nuclei, locus coeruleus, and NTS; orexin increases inspiratory capacity [29,37].

Fastigial nucleus (cerebellum) contains glutamatergic projection neurons, with efferents also toward NTS; when activated, perhaps by chemical imbalances (hypercapnia) or mechanical alterations of the respiratory trunk, it implements the inspiratory force and respiratory rhythm [38]. The cerebellar cortex sends efferents to the Kölliker-Fuse nuclei, but many indirect connections exist for the stimulation of the most important respiratory centers; activation of the cerebellar cortex occurs when the pressure of the surrounding air changes, in the presence of hypoxia and hypercapnia and with voluntary expiration [29].

Paratrigeminal nucleus (interstitial system of the spinal trigeminal tract) is located in the dorsal lateral medulla; it is able to send efferents to NTS, parabrachial nuclei, Kölliker-Fuse nucleus. About 72% of the neurons that constitute this portion of the medulla are barosensitive; it is activated by the action of cough/apnea [39,40]. Spinal trigeminal nucleus sends projections toward preBötzC, Kölliker-Fuse nuclei, parabrachial nuclei, NTS, cVRG. Its activation occurs to reduce the force of inspiration and during actions related to breathing; it also receives afferents from the olfactory nerve (from the nasal mucosa), from trigeminal endings from the nasal epithelium, without passing through the olfactory system, to stimulate sniffing [29,41].

Reticular formation (a circuit that resides between the brainstem, medulla oblongata, pons), in experimental animal model, seems to positively influence the phrenic nerve, probably with premotor function [29]. The central and lateral amygdala sends efferents to preBötzC, NTS, locus coeruleus, retrotrapezoid nucleus, parabrachial nuclei with inhibitory effects [29,42,43].

Bed nucleus of the stria terminalis (extension of the amygdala) intervenes to regulate breathing during emotional states of anxiety and fear, slowing down its rhythm, with direct connections to the NTS and parabrachial nuclei [43].

The cerebral cortex (primary motor, premotor and supplementary motor area) has direct connections with the phrenic nuclei, bypassing the most important respiratory centers, with direct efferents also toward preBötzC, Kölliker-Fuse nuclei and NTS; the cortical impulse has excitatory effects [43].

The paraventricular nucleus (hypothalamus) has direct connections with all the most important respiratory areas mentioned in this paragraph, with excitatory activity, including the phrenic nuclei with increased diaphragmatic activity [29,43]. The perifornical area (hypothalamus) directly involves NTS, Kölliker-Fuse nucleus, parabrachial nuclei, managing breathing based on present emotions, such as fear and anxiety [43,44]. The lateral and dorsal area of the hypothalamus influences the modality of the respiratory rhythm, through direct connections to Kölliker-Fuse, parabrachial nuclei, rVRG; these hypothalamic portions influence breathing in the presence of stress and during sleep, respectively [43].

The thalamus sends efferents to the rVRG area, and depending on the emotional or postural condition, or sleep and wakefulness, it can stimulate or slow the respiratory rate [43].

The periaqueductal gray area (mesencephalon) projects nerve endings to cVRG, NTS, Kölliker-Fuse, preBötzC, PiCo, retrotrapezoid nucleus; this connection has the purpose of modulating the muscles involved in vocalization, in particular, in the presence of emotions (laughing or crying) [29].

The pedunculopontine tegmental nucleus (dorsal pons) is important for managing breathing during sleep; it projects to retrotrapezoid nucleus, Kölliker-Fuse [29,45].

Basal ganglia (telencephalon/mesencephalon) directly send efferents to preBötzC. This connection probably influences the respiratory rhythm [46,47].

Spinal areas can influence the rhythm and pattern of breathing. Spinal connections of “pre-phrenic” neurons or interneurons can change synaptic contacts depending on the stimulation present (chemical, mechanical, etc.), involving motor neurons of the respiratory muscles in a different way, generating rhythmogenic stimuli, as they are able to stimulate the nuclei of the phrenic nerves. In addition, spinal neurons dedicated to the muscles of the limbs can, in some circumstances, create respiratory rhythms, stimulating the phrenic nuclei. Locomotor activity can drive the respiratory rhythm from neurons located in the spinal cord (propriospinal neurons); this event allows a maximum coordination between breathing and movement (“spinal respiratory rhythms”).

**Figure 1:**
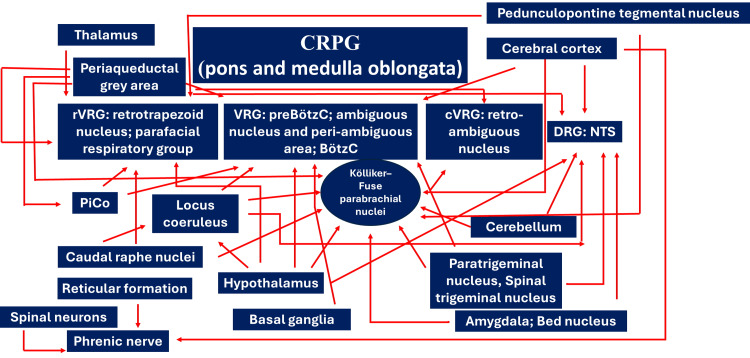
The simplified diagram highlights the most important efferent connections with the central respiratory pattern generator and other neural areas (details are in the text). Diagram created by Bordoni Bruno. BötzC: Bötzinger complex; CRPG: central respiratory pattern generator; cVRG: caudal ventral respiratory group; DRG: dorsal respiratory group; NTS: nucleus of the solitary tract; PiCo: post-inspiratory complex; preBötzC: pre-Bötzinger complex; rVRG: rostral respiratory group; VRG: ventral respiratory group

This mechanism may occur simultaneously with higher orders arriving at the phrenic neuron pool. Most likely, this response between locomotion and breathing is facilitated by stimulation of peripheral receptors (proprioception, nociception, etc.), which may send afferents to the NTS, the spinal trigeminal nucleus and also to spinal interneurons for the motor neurons of the musculature. This occurs with every breath. Peripheral muscle weakness may underlie weakness of the diaphragm muscle [28,48]. Some propriospinal neurons may be directly driven by rVRG, while others may exhibit phasic bursting [49]. Adequate breathing allows for adequate neuromuscular expression [28,49]. Activation of propriospinal neurons may activate spinal neurons serving the accessory respiratory musculature, for example, during sustained physical activity (Figure 1) [49].

The following paragraph will list the respiratory accessory muscles of the skull area, to continue with the description of other main and accessory muscles related to breathing in the subsequent sections of the text. When present in the literature, information related to the clinical setting will be added.

Respiratory musculature of the skull

The neural network that manages breathing, in a healthy subject with eupneic respiratory acts, must ensure about 700 breaths in an hour. Breathing can be divided into phases: pre-inspiration (before the intervention of the diaphragm muscle); inspiration (inspiratory muscles); post-expiration (relaxation of the diaphragmatic contraction); post-inspiration (forced inhalation); post-expiration (forced exhalation and actions such as coughing and sneezing, etc.) [50].

The first pair of muscles that activates for the pre-inspiration phase is the alae nasi or dilator naris, which maintain upper airway patency [50]. It is an inspiratory muscle and is used to understand the degree of dyspnea (using electromyographic activity) in patients with a critical clinical picture [51,52]. It activates before the complete contraction of the diaphragm (100-150 milliseconds before) [53]. The activity and electrical magnitude of these muscles depend on the air resistance recorded by the central nervous system; furthermore, the shift of activation to the oronasal areas allows to minimize the pulmonary resistance in this phase [54]. During nose breathing and with chewing present (closed mouth), the respiratory rate increases, while the movement of the rib cage decreases. However, if there is a nasal obstruction, with the presence of the bolus/water preparation in the oral cavity, the strength and duration of dental contact decreases [55]. It also slows down the respiratory rate and increases the movement-amplitude of the rib cage compared to nasal breathing [55].

The levator labii superioris alaeque nasi muscle is lateral to the nose, is activated for facial expressions, and by contracting simultaneously widens the nostrils; probably, they are activated for a forced inspiration, but we have limited data [56]. Likewise, the procerus or pyramidalis nasi muscle intervenes sporadically in inspiration (root of the nose), probably for a forced inspiration [56].

Musculus depressor septi nasi dilates the external nasal valve and prevents collapse of the nostrils, improving the entry of the air flow in inspiration [57].

The nasalis muscle (alar and transverse parts) or compressor nasi is activated before the activation of the diaphragm muscle and is part of the pre-inspiration mechanism; it contributes to maintaining the opening of the nostrils during a forced inspiration [56,58,59].

The apicis nasi or small compressor narium minor muscle helps keep the airway open during a forced inspiration [59].

The anomalous nasi muscles are deep (under the transverse nasalis muscle) and are activated by a forced and prolonged inspiration, helping the constant entry of air [59].

Orbicularis oris is activated when nasal resistance to the passage of air is increased [60].

When the nasal muscles have an adequate physiological tone, the ability to widen the nostrils improves, improving the capacity for air to enter during inspiration [61]. Evaluating and knowing the function of these muscles is essential to correctly frame the therapeutic approach, for example, focusing attention on the nose [61]. When a supine position is assumed during sleep, nasal airway resistance increases (an increase in venous pressure of up to 8 mmHg can occur, which causes an increase in nasal congestion). During deep sleep with nonrapid eye movement, the phenomenon of hypoventilation (physiologically or pathologically) is more pronounced, which is the sum of a decline in CRPG activity and an increase in airway resistance [62]. In healthy subjects, nasal breathing in the supine position and during sleep is present; it is important to evaluate the function of the muscles to understand the behavior of the nasal muscles responsible for dilating the nostrils. Nasal obstruction can lead to sleep apnea syndromes, hypoxia with negative cognitive consequences [62]. Zygomaticus minor muscle connects to nasalis muscle (wing portion) to help the latter in keeping the nasal airway open (Table 1) [63].

**Table 1:** Summary of the musculature of the skull involved in breathing and that is managed by the respiratory centers Table created by Bordoni Bruno.

Respiratory muscles of the cranium
Alae nasi
Levator labii superioris alaeque nasi
Musculus depressor septi nasi
Nasalis
Apicis nasi
Anomalous nasi
Orbicularis oris
Zygomaticus minor

Eighty percent of patients suffering from OSAS have nasal airway obstructions; equally, nasal airway obstruction can cause cardiac arrhythmic problems and sudden death over time [64,65].

The following paragraph will list the respiratory musculature of the bucco-cervical area, inserting more relevant clinical information related to the musculature mentioned.

Bucco-cervical area

Mylohyoid muscles (floor of the mouth, hyoid bone and mandible) are activated when nasal resistance increases, thanks to the activation of mechanoreceptors found in the oropharyngeal airway [60]. They intervene in phonation, respiration, swallowing and chewing, acting on the position of the hyoid bone [66,67].

The masseter muscle (masticatory muscle) intervenes when inspiratory resistance increases, preceding the decrease in pharyngeal pressure, like the muscles of the nose; during sleep, the mandible moves with an electromyographic spectrum of the masticatory muscles that mirrors the diaphragm [55,68,69]. Mandibular movements during sleep are predictive for the detection of sleep apnea disorders [69]. The activation of the masseter by respiratory stimuli is concomitant with the lingual complex; the resulting mandibular movement allows for better functioning of the tongue during breathing [70].

The temporalis muscle (masticatory muscle) is activated more for nasal breathing than for oral breathing; this muscle probably mirrors the behavior of the masseter muscle, as described in this paragraph [71].

The pterygoid muscles (masticatory muscles) are activated to assist oral breathing by moving the jaw and by reflex to assist the movement of the tongue during breathing; if the masticatory muscles are not consistently active during sleep, more apnea episodes develop [72,73].

There are no precise data on the buccinator muscle and its role in breathing, except anecdotally.

The digastric muscle (helps to open the jaw and bring the hyoid bone posteriorly) is influenced by CRPG during swallowing and breathing, probably to allow the tongue to perform its functions at its best; this mechanism is recorded especially with nasal breathing [74-76].

Another muscle of the suprahyoid group and which intervenes in the mechanisms of breathing is the geniohyoid. The latter is electrically activated (little) during a eupneic act and especially during expiration; it plays an important role in avoiding dysphagic problems [77].

The stylohyoid muscle, part of the suprahyoid group and agonist of the geniohyoid muscle, contracts to manage the hyoid movement during swallowing and breathing, in concert with the lingual complex; furthermore, it contracts to counteract the negative pressure during inspiration [78].

The last suprahyoid muscle is the mylohyoid, which contracts to allow adequate swallowing, preventing the entry of the bolus into the upper airways during chewing/breathing [79]. It is important for correct control of breathing and for phonation through control of hyoid movement [66,80].

The suprahyoid muscles are important to avoid dysphagic, phonatory, and respiratory dysfunctions; in chronic pathologies, such as COPD and CHF, a high percentage of phonatory problems (these reflect a poor ventilatory capacity) and dysphagic problems (these are related to a respiratory dysfunction) are found [81-85].

The levator palatini muscle is activated with oral breathing, and with incoming/outgoing airflows not at rest; it probably plays a role in the management of air pressures [86,87].

The platysma muscle intervenes during forced inspirations and very little during eupneic breathing; if the muscle is weak or sarcopenic, it can become a cause of dysphagia [88,89]. Observing myofascial bands anteriorly and superficially to the neck could indicate hyposthenia and sarcopenia of this muscle, with alterations of the respiratory and swallowing mechanism.

The infrahyoid muscles facilitate the flow of air during inspiration [90]. The sternothyroid is activated in the early phases of inspiration to keep the upper airways open, counteracting negative pressure; its dysfunction could aggravate the symptoms of sleep apnea [91-93]. The sternohyoid is a muscle involved in the act of breathing, in addition to swallowing and phonation, like all the infrahyoid muscles [74]. The sternohyoid depresses the hyoid bone and is activated for inspiration; during the night hours, it constantly contributes to keeping the upper airways open during the phases of inspiration, with optimization of diaphragmatic contraction, whereas its dysfunction could aggravate the apneic symptoms [91,94,95]. The thyrohyoid muscle is activated to facilitate the entry of air into the upper airways and intervenes more if there is a restriction that prevents the correct incoming airflow [94]. The omohyoid muscle depresses the hyoid bone for inspiration, keeping the upper airways open, and like all the other infrahyoid muscles helps the function of the tongue and swallowing (it also contracts when the shoulder is abducted to 90 degrees) [96]. During a deep inspiration, the contraction of this muscle allows to keep the lung apex in place (preventing the collapse of the lung structure) [97]. Chewing, swallowing, and breathing are all actions that must necessarily be integrated by CRPG; observing a dysfunction of the suprahyoid and infrahyoid muscles means understanding how these functions interact.

The suboccipital musculature is indirectly involved in respiratory function. If this area is dysfunctional (painful, hypomobile, hypotrophic, etc.) there is a decline in lung volumes and a reduction in the strength of the most well-known respiratory muscles, and a reduction in the capacity for thoracic movement [98,99]. In a clinical setting of respiratory dysfunction, this anatomical area must be evaluated and possibly become the target of curative approaches (physiotherapy, manual medicine, pharmacology, etc.) [98,99]. This dysfunctional context between suboccipital musculature and lung volumes is even more easily found in patients with persistent mouth or oral breathing [100].

The trapezius muscle is activated during forced inspirations, in the presence of nasal airway obstruction, or when breathing through the mouth while chewing; its contraction reflects the increased movement of the rib cage during inspiration [55]. The respiratory centers activated during walking also manage the trapezius muscle (and the sternocleidomastoid (SCM)), ensuring better neuromotor control during walking [101]. Similarly, if inspiration needs to be increased in volume and force, the trapezius muscle, both in the erect and supine position, is activated to a greater extent than the SCM and the scalenes [102].

The SCM muscle intervenes in case of forced inspirations, helping to expand the volume of the rib cage, in particular with values over 50% of the maximum subjective inspiratory force (MIP), where diaphragmatic activity tends to decrease at the same time [101-103]. Evaluating the SCM allows us to understand the functional capacity of the diaphragm; up to 50% of the MIP diaphragm is the main muscle activated with low myoelectric activity of the SCM, beyond which percentage, the ratio is reversed. If there is a respiratory dysfunction (COPD, acute respiratory failure), the activity of the SCM is found significantly with greater electrical activity (the diaphragm always intervenes, but with less force than the force recorded by the SCM), becoming a predictive event of pulmonary pathology [104].

The scalene muscles are always active during inspiration and increase their activation when the load of inspiration (increased resistance) increases; they facilitate the movement of the thorax by lifting the first two ribs during inspiration [105]. In case of respiratory pathologies, these muscles undergo shortening and thickening with an inverse ratio compared to the diaphragm muscle (hypotrophy); the greater the diaphragm dysfunction, the greater the activation of the scalene muscles [105].

The palatoglossal and palatopharyngeal muscles, which are part of the soft palate, are electrically activated in a rhythmic manner together with the alae nasi muscle, the lingual complex and in temporal coordination with the diaphragm [50]. They depress the soft palate during inspiration, widening the upper airway for air entry and assist in the function and movement of the tongue; functional alterations of these muscles determine the recurrence of sleep apnea [50,106].

The tensor palatini is also part of the muscles that make up the soft palate and is activated during inspiration/expiration; the levator veli palatini muscle (soft palate) is activated during expiration/inspiration, with greater activity when breathing with the mouth open. These two muscles, active during the phases of breathing (inspiration and expiration), act to close the retropalatal oropharynx, particularly during oral breathing and swallowing [50,106]. The last muscle of the soft palate is the musculus uvulae, for which we have no certain data regarding its respiratory function; it probably helps the entry of air from the nose [50,106].

The pharyngeal constrictor muscles (superior or glossopharyngeus, middle, inferior) behave by mirroring the lung volumes; if the lung volumes are high, these muscles constrict, while if the lung volumes are low, they relax and dilate the upper airways [106].

The stylopharyngeal, salpingopharyngeal, and palatopharyngeal muscles (pharyngeal muscles) maintain the correct tone of the pharynx during the passage of air (inspiration), counteracting its collapsibility [50].

The cricothyroid and posterior cricoarytenoid (laryngeal muscles), are activated during inspiration to keep the upper airway open, particularly with forced inspirations [50]. Thyroarytenoid, interarytenoid, and cricothyropharyngeus muscles (laryngeal muscles) help the cricoid cartilage move in the horizontal plane to facilitate the entry of air into the lungs during inspiration [50].

The lingual complex is a coordinated group of intrinsic and extrinsic muscles with very complex and diverse tasks, both local and systemic, including allowing correct breathing. The extrinsic (genioglossus, styloglossus, hyoglossus, palatoglossus) and intrinsic (transversalis, inferior, and superior longitudinalis, verticalis) muscles are activated in different ways to allow the tongue to act correctly; it is preferable to consider the tongue as a lingual complex and not as the expression of single muscular structures [107-109]. During inspiration, the lingual complex contracts inferiorly and pushes the hyoid bone forward, so as to keep the upper airways open; when expiration occurs, the tongue is relaxed, and the upper airways are closed [107-109]. The chondroglossus muscle is another extrinsic muscle of the lingual complex, not always considered, which starts from the lesser cornu of the hyoid bone to merge with the intrinsic (inferior longitudinal muscle) and extrinsic (genioglossus muscle) musculature [110]. It probably acts to assist the lingual complex during lingual functions, including inspiration (Table 2) [110].

**Table 2:** Summary of the musculature of the bucco-cervical area that is involved in breathing and is managed by the respiratory centers Table created by Bordoni Bruno.

Respiratory muscles of the bucco-cervical area
Mylohyoid
Masseter
Temporal
Pterygoids
Digastric
Suprahyoids
Infrahyoids
Levator palatini
Platysma
Suboccipital muscles
Trapezius
Sternocleidomastoid
Scalene muscles
Soft palate muscles
Pharyngeal muscles
Laryngeal muscles
Lingual complex muscles

Cervicothoracic area

Given the width of the trapezius muscle, let us reconsider this muscle in the cervicothoracic area. The trapezius muscle during a forced inspiration is activated to allow lifting of the scapulae and rotation of the clavicles, allowing the SCM to lift the anterior-superior thoracic area more easily. The myoelectric intervention of the trapezius in the supine position is greater, always during a forced inspiration, than the scalene musculature and the SCM [111]. In a bedridden patient, evaluating and paying attention to the function of the trapezius muscle becomes important to help functional recovery. If the patient is an oral breather, not only is the diaphragm activated to a lesser extent, but also the accessory muscles such as the trapezius undergo less activation [112].

The pectoralis major muscle falls into the group of respiratory accessory muscles, and as such, this muscle can be managed by CRPG [113]. The pectoralis major is active both during inspiration (to help move the ribs) but also during coughing (forced expiration); the greater the reduction in lung capacity, the lower the strength expressed by this muscle, which becomes a predictor of mortality and morbidity in respiratory diseases [114-116].

The pectoralis minor muscle is involved in forced inspiration by helping to lift the ribs; its strength and volume are directly proportional to the decrease in lung volumes and becomes, as for the pectoralis major muscle, a predictive area of mortality and morbidity, even in patients not necessarily with respiratory diseases [115,117,118].

The latissimus dorsi muscle is activated linearly with increasing effort during inspiration by lifting the lower ribs, and a direct relationship is found between the volume of this muscle and the severity of the respiratory disease [119]. It can also intervene in coughing and sneezing [120].

The serratus anterior muscle acts to help lift the ribs or maintain their position based on the posture assumed by the person, thus acting as an aid in inspiration or expiration [121,122].

The serratus posterior superior muscle is activated to assist in inspiration by lifting the ribs, while the serratus posterior inferior muscle is activated to facilitate expiration with the opposite action; these statements, however, do not find full agreement in the literature [123,124].

The levator scapulae muscle intervenes to lift the scapula during inspiration, and in case of weakness or pain, the patient may complain of dyspnea with the reduction of thoracic volumes; this clinical event can also occur for other muscles that are involved in scapular and costal movement, such as rhomboids, teres major and minor, and infraspinatus [125].

The subclavius muscle elevates the first rib by intervening during a forced inspiration; its dysfunction could be the expression of diaphragmatic suffering (Table 3) [126,127].

**Table 3:** Summary of the muscles involved in respiratory acts in the cervical-thoracic area Table created by Bordoni Bruno.

Respiratory muscles of the cervical-thoracic area
Trapezius
Pectoralis major
Pectoralis minor
Latissimus dorsi
Serratus anterior
Serratus posterior superior
Serratus posterior inferior
Levator scapulae
Rhomboids
Teres major and minor
Infraspinatus
Subclavius

The following paragraph will list the respiratory musculature of the thoracic area, adding clinical information when relevant.

Thoraco-abdominal area

The erector spinae muscles are activated during forced expiration and Valsalva maneuver and demonstrate a direct relationship between the loss of tone/volume in mechanically ventilated patients and the clinical respiratory severity, mortality rate, and length of hospital stay of patients; similarly, the decrease in volume/strength of these muscles reflects the mortality/morbidity rate in patients with COPD [128-130].

The quadratus lumborum muscle intervenes in the phases of inspiration, probably to support the function of the diaphragm, but the data we have are very limited [131]. We also have no data on the functional condition of this muscle and the pathologies that may involve the mechanisms of breathing.

The levatores costarum muscle is activated to influence the movement of the ribs during non-forced inspiration [132]. Probably, in patients with COPD in an advanced stage and with the Hoover's sign, these muscles could be hypotonic/hypotrophic, but we have no certain data.

The transversus thoracis (also known as triangularis sterni, sternocostalis) is activated during the expiration phase [133]. We have no data on adaptation in the presence of respiratory pathologies or in frail patients.

The external intercostal muscles contract during inspiration to bring the ribs outward and into external rotation (small movements, almost vibrations, which together "open" the rib cage), while internal intercostal muscles act during expiration in a contrary costal manner, although a caudal portion of these latter muscles can be activated for the start of inspiration [134,135]. The cranial portion of the intercostal muscles has a greater mechanical advantage and contracts more rapidly [49]. In the presence of chronic respiratory diseases, the internal and external intercostal muscles undergo a significant decline in volume and strength, reflecting the severity of the disease (lung volumes, spirometry), with fat infiltration; similarly, in chronic cardiovascular diseases these muscles are hypotonic and deoxygenated [136-138].

The diaphragm muscle, the main muscle for inspiration, performs several tasks for bodily well-being, and its dysfunction creates various pathological conditions [1]. When it contracts for inspiration, it pushes the ribs laterally and downward, and to a very limited extent the sternum and ribs anteriorly; in this way, it decreases thoracic pressure and promotes airflow [139]. After activation of the phrenic nerve, it takes about eight milliseconds before the diaphragm contracts during inspiration [53]. In healthy women, there is a greater resistance to contractile fatigue before reaching fatigue, compared to healthy men; the latter use the accessory muscles in a greater percentage to compensate for the finding of diaphragmatic fatigue [140].

The abdominal muscles, such as the internal and external oblique, the transverse abdominis and rectus abdominis, act for forced expiration, where they promote the propulsive force toward the outside (coughing, sneezing, vomiting) [141]. When the diaphragm descends, the abdominal muscles decrease their tone, to offer less resistance to the descent of the diaphragm during inspiration [28]. If the abdominal muscles are dysfunctional, the diaphragm will have difficulty creating the right intrathoracic and abdominal pressures, as in patients with COPD, with a detriment to the symptoms [142]. There is an altered intervention pattern for the abdominal muscles in patients with COPD, where the rectus abdominis and the external oblique intervene with a greater entity than the deeper abdominal muscles (transversus abdominis and internal oblique), probably due to muscular weakness and central dyscoordination [142]. In patients with OSAS, an early and uncoordinated intervention of the abdominal muscles with the diaphragm muscle is recorded, making the apneic symptoms more important; furthermore, a decrease in muscle volumes negatively affects the parameters of apnea [143,144]. Evaluating and considering abdominal muscles allows us to identify possible respiratory dysfunctions (Table 4).

**Table 4:** The muscles involved in breathing for the thoracic area Table created by Bordoni Bruno.

Respiratory muscles of the thoraco-abdominal area
Erector spinae muscles
Quadratus lumborum
Levatores costarum
Transversus thoracis
Intercostal muscles
Diaphragm
Abdominal muscles

Pelvic area

To try to understand how to improve breathing in a clinical and/or sports setting, it is also necessary to know the muscles involved in the systemic act of breathing. The muscles that make up the pelvic floor are considered accessory respiratory muscles. When inhaling, the pelvic muscles relax to allow the diaphragm to descend more easily and create greater intra-abdominal pressure [25,127,145]. The pelvic muscular area must be considered to better understand how the mechanisms of breathing work [146]. Urinary incontinence is an independent predictor of the occurrence of chronic lung diseases such as in patients with COPD [147,148]. Over half of patients with CHF complain of incontinence problems [149]. The pelvic muscles express greater force during expiration or acts related to breathing such as coughing or sneezing [150]. Training the pelvic floor muscles has a positive effect on increasing lung volumes, as well as a better parasympathetic response helping cardiovascular function, and better management of fluid pressures (blood, lymph, cerebrospinal fluid) [127,151,152].

The pelvic musculature includes the levator ani (puborectalis, pubococcygeus, iliococcygeus) and ischiococcygeal; also, to be considered are muscles that are activated when the pelvic floor is activated and that have direct correlations with the levator ani, such as the gluteus maximus, or indirect relationships such as the obturator internus (via the obturator fascia) [153-155]. Another muscle that is activated simultaneously with the pelvic floor is the piriformis [156].

We do not have precise and more specific data on each muscle portion that constitutes the pelvic complexity with respect to respiratory functions (Table 5).

**Table 5:** The muscles that intervene in breathing for the pelvic floor area Table created by Bordoni Bruno.

Respiratory muscles of the pelvic floor muscles
Levator ani
Ischiococcygeal
Gluteus maximus
Obturator internus
Piriformis

## Conclusions

The narrative review reviewed all the muscles that are involved in the breathing mechanism and that are controlled and managed by the respiratory centers, starting from describing the cranial area up to the pelvic area. To have a comprehensive clinical picture, it is necessary to have all the anamnestic data of the patient, and so it is necessary to know all the muscles that participate in breathing to understand how to obtain a comprehensive approach for the care and treatment of the patient in improving their breathing capacity. In the osteopathic field, knowing the muscular areas involved in breathing is useful for translating the palpatory sensations derived from the muscles into manual terms, so as to better subjectivize the chosen manual therapeutic approach. Several more specific and precise data on the function of some muscles are missing, and research should make a further effort to deepen the gray areas still present, with the goal of implementing the healing approach to the person. This is the first article in the literature, to the knowledge of the authors, that tries to include in a single text all the muscles directly or indirectly involved in breathing.
